# Imagining a better world: assessing the immediate and delayed effects of imagined contact on attitudes toward refugees in elementary school

**DOI:** 10.3389/fpsyg.2024.1294208

**Published:** 2024-04-29

**Authors:** Antonija Vrdoljak, Dinka Čorkalo Biruški, Nikolina Stanković, Rachel Fasel, Fabrizio Butera, Margareta Jelić

**Affiliations:** ^1^Faculty of Humanities and Social Sciences, University of Zagreb, Zagreb, Croatia; ^2^Faculty of Social and Political Sciences, University of Lausanne, Lausanne, Switzerland

**Keywords:** imagined contact, refugee integration, school intervention, long-term effects, intergroup attitudes, contact intentions

## Abstract

**Introduction:**

Preparing host-society children for contact with refugees coming into their classes poses a new and important challenge for countries with little prior experience in integration. Imagined contact is a prejudice-reduction intervention that can be particularly useful in this context. However, its long-term effects and potential age-related variations in its efficacy among primary school children remain understudied.

**Methods:**

This study investigated the short-term and long-term effects of an imagined contact school intervention on the change in attitudes and contact intentions of 1,544 children aged 7–15. Of these, 827 participated in a four-session-long intervention delivered by their teachers within their regular classes, while 717 served as a comparison group. Short-term effects were assessed approximately one week after the last intervention session, with long-term effects evaluated around two and a half months later.

**Results:**

Our findings indicate that the imagined contact intervention instigates positive changes in intergroup attitudes and contact intentions in both the short term and long term, but only for the children in the lower grades of primary school.

**Discussion:**

While the durability of these effects among younger participants holds promise for future use of imagined contact in schools, we also scrutinize potential developmental and methodological explanations of the absence of expected intervention effects among older children.

## 1 Introduction

Prejudice toward refugees not only poses serious challenges to the well-being and integration of refugees but also hinders the social fabric of host communities. As the global refugee crisis continues to escalate, it becomes crucial to address this pressing concern through evidence-based interventions. Schools are the best place to implement interventions aimed at reducing prejudice toward refugees because they provide a nurturing and inclusive environment where students can learn, interact, and gain a deeper understanding of diverse cultures and experiences.

This study aims to shed light on the importance of researching the effectiveness of prejudice-reduction interventions in Croatia, a small European country with no prior experience in refugee integration. Due to this inexperience, Croatian schools lack the means and developed procedures to address the challenges connected to refugee integration. At the time we conducted this study, the number of refugees in the country was still relatively small, and most of the refugee children came from Middle Eastern countries. The arriving children were situated in only several schools in four different cities, meaning that most Croatian children have never had the opportunity to meet a refugee child. However, the geopolitical situation worldwide, as well as international agreements pertaining to the relocation of refugees on the basis of European solidarity and Dublin III regulation, suggested that the issue of refugee integration could become more pressing in the coming years. Qualitative studies conducted in Croatian schools attended by refugees have shown that, while instances of negative contact and discrimination against refugee children are rare, positive contact between refugee children and their peers is also not common (Perić and Merkaš, 2020; Vrdoljak et al., 2024). Furthermore, children are usually not prepared for the arrival of a refugee peer in their classes, which sometimes leads to reluctance to accept and socializing with a refugee child (Vrdoljak et al., 2024). With the rising number of refugees in Europe, especially after the outbreak of the Russian invasion of Ukraine, the need to prepare children for positive initial interactions with refugee peers becomes even more evident.

Furthermore, from a developmental standpoint, school age is the most appropriate period for the implementation of interventions aimed at prejudice reduction. According to the social-cognitive approach to prejudice development, after approximately the age of seven, children enter the stage of concrete operations and develop the cognitive capacities necessary to participate in prejudice-reduction interventions (Aboud and Steele, 2017). The theory of social identity development considers this period to be crucial for the emergence of negative attitudes but also for the possibility of acting on children’s prejudices (Nesdale, 2004). After the age of seven, children who strongly identify with their group, whose group endorses norms of negativity toward the outgroup, who perceive the outgroup as a threat to the ingroup, or assume that the ingroup will profit from the expression of a negative attitude, may enter a phase of negativity toward the outgroup. This can lead to different patterns of prejudice development for different children during and after middle childhood, and it also suggests that children’s attitudes during school age can change under the influence of parents, teachers, and peers, as well as specific interventions (Nesdale, 2004). Typically, school interventions aimed at promoting intergroup contact rely on well-confirmed Allport’s contact hypothesis (Allport, 1954), which assumes direct positive intergroup contact as necessary to improve intergroup attitudes and consequent behaviors. Nevertheless, more contemporary developments in the contact hypothesis have proved that using indirect contact (e.g., Vezzali et al., 2015d) or even imagining positive intergroup contact can aid in achieving the same goal. Imagined contact refers to “a mental simulation of interaction with a member or members of an outgroup” (Crisp and Turner, 2009, p. 234). The use of this method does not require the real presence of an outgroup member, and it can be used even when there have been no instances of prior direct contact with an outgroup (Crisp and Turner, 2012). The intervention is often regarded as a pre-contact tool, and when the characteristics of the outgroups were explained before the imagination task, it was effectively applied with children without direct contact experiences (Vezzali et al., 2020; Ginevra et al., 2021). Therefore, imagined contact can be used before the inclusion of refugee child(ren) in the class as a preparatory and preventative activity which could foster a positive initial attitude toward refugee classmates and prepare host-society children for direct intergroup contact.

In an imagined contact intervention, participants are first presented with a scenario in which a situation of contact with an unknown outgroup member is described and then asked to continue imagining that meeting by themselves for several minutes. The period of imagining can be followed by some form of “reinforcement” of the effect of imagining, such as writing, drawing, discussing, or retelling the imagined content (Crisp and Turner, 2009; Miles and Crisp, 2014). The main goal of imagined contact studies is usually focused on the change in intergroup attitudes, and most evaluations of imagined contact include at least one measure of attitudes. The effect of imagined contact on attitudes has been well established in a meta-analysis, which yielded an effect size of *d* = 0.36 for measures of explicit attitudes (Miles and Crisp, 2014), and it is also often found in studies conducted with children (e.g., Cameron et al., 2011; Birtel et al., 2019; Vezzali et al., 2020; Ginevra et al., 2021).

Furthermore, in the context of preparing host-society children for future interactions with refugees, their intention for contact is a particularly important outcome. Contact intention refers to children’s readiness to engage in interaction with a refugee child, their openness to intergroup friendships, and their preparedness to welcome a refugee child into their class. Previous research has shown that both children and adults who imagined intergroup contact reported more positive intentions for contact with an outgroup member (e.g., Husnu and Crisp, 2010; Cameron et al., 2011; Birtel et al., 2019). Meta-analysis of imagined contact effects has confirmed these effects, yielding an effect size of *d* = 0.46 for the measures of behavioral intentions (Miles and Crisp, 2014).

Studies of imagined contact conducted with children have produced promising results and highlighted the potential for school intervention development (Crisp and Turner, 2012), showing stronger effects than those conducted with adults (Miles and Crisp, 2014). However, the meta-analysis included a small number of studies conducted with children and adolescents, and some more recent studies were not able to replicate the effects of imagined contact on the measures of attitudes and contact intentions (e.g., Fleva, 2014; Mazure, 2016; West and Greenland, 2016; Kinghan, 2019; Constantin and Cuadrado, 2021), Study 1; see also studies by Smith and Minescu (2022a) and Vezzali et al. (2015c), which found significant effects only for some varieties of imagined contact.

In two separate studies, Smith and Minescu (2022a, 2022b) demonstrated the efficacy of an enhanced, norm-framed imagined contact intervention executed within a typical school classroom. Their studies highlighted the intervention’s potential to enhance attitudes and intentions for contact with refugees among primary school children in Ireland. The first study found the immediate effects of norm-framed imagined contact on intergroup warmth and stereotyping bias. However, this was not established for a standard imagined contact procedure, which did not differ from a control group (Smith and Minescu, 2022a). The subsequent study revealed that the impact of norm-framed imagined contact on contact intentions persisted for a two-week period following the intervention, although this effect was not observed for the measures of intergroup warmth and stereotyping bias (Smith and Minescu, 2022b). The latter study also lacked a control group, focusing on the comparison between an enhanced norm-framed scenario and the standard imagined contact scenario instead.

The present study aims to expand upon the findings of previous research by investigating the effectiveness of an imagined contact intervention conducted within a real school environment. Our approach involved teachers facilitating the intervention, encompassing the entire class simultaneously and incorporating a mix of collective class-level activities and individual tasks. We believe that employing intervention in such a way is necessary for establishing the external validity of imagined contact and transitioning from tightly controlled laboratory settings to more practical, real-world scenarios. The intervention proposed in this study is designed for easy integration into daily school routines and can be seamlessly incorporated into curriculums with minimal adjustments. This study also included a larger sample, spanning various age groups across the entire primary school spectrum and situated within a country with limited experience in refugee integration, where preparatory interventions like the one examined are particularly important. Moreover, we have extended the follow-up period significantly beyond the time frame observed in prior studies.

Examining the duration of imagined contact effects is one of the most frequently mentioned challenges in the field. If the results reflected only short-term changes, despite all the advantages, this intervention would be less significant as a part of prejudice-reduction programs (Brown and Paterson, 2016). A couple of longitudinal studies conducted with students and adults suggest that the effects might persist for some time after the intervention—from 1 month (Falvo et al., 2014) up to 7 months (Vezzali et al., 2015a). However, most imagined contact studies include much shorter post-test periods.

In studies conducted with children, researchers typically examine the effects of imagined contact either immediately after the intervention (e.g., Smith and Minescu, 2022a) or 1 week later (e.g., Vezzali et al., 2012a, 2012b, 2015b, 2020; Stathi et al., 2014). However, some studies have found effects persisting up to 2 weeks after the intervention (Vezzali et al., 2015c; Ginevra et al., 2021; Smith and Minescu, 2022b). While the persistence of effects for two weeks suggests that they are more than just immediate reactions to imagined scenarios, it is difficult to consider them genuinely long-term effects. Few studies that examined longer time lags (three to seven months) were unable to detect imagined contact effects (Mazure, 2016; Kinghan, 2019). However, it is important to note that neither of these studies succeeded in eliciting effects even during immediate post-tests. To provide more information on the issue, this study included two post-test observations—one examining short-term (approximately 1 week after the last intervention session) and one long-term effects of imagined contact (approximately two and a half months after the last session).

The goal of this study is to provide schools with an effective, widely applicable and easily adaptable imagined contact intervention. However, results of a recent systematic review suggest that imagined contact interventions conducted with younger children might be more effective than those conducted with children older than 11 (Vrdoljak et al., 2023). These differences in intervention effectiveness could stem from differences in developmental stages, or they could point to differences in methods used. Namely, interventions conducted with children over 11 are often more similar to those conducted with adults; they usually include fewer sessions of imagining contact and less creative scenarios and reinforcement techniques. Therefore, we examine the effects on younger and older children separately. We tried to provide different age groups with engaging and age-appropriate intervention activities, which would include only minimal adaptations for different age groups, expecting that this approach would prove appropriate for both older and younger children.

The division of children into younger and older age groups follows the education system worldwide, and here we briefly describe the system in the current research context, i.e., the schools in Croatia. Primary education in Croatia is organized into eight grades, divided into two stages. The first stage consists of grades one to four (children aged from 6 to 10). They are commonly referred to as the lower grades, and each class is taught by a single teacher who covers various subjects. In contrast, the upper grades include grades five to eight (children aged from 11 to 15). In these grades, specialized teachers are assigned to each subject. These teachers instruct different classes in the same subject, with their expertise primarily focused on the subject matter rather than on child development and education practices.

To summarize, in this study, we focus on both short-term and long-term effects of imagined contact intervention on two dependent variables—attitudes toward refugees and intention of intergroup contact. To assess the effectiveness of imagined contact interventions, it is important to explore outcomes that align closely with the goals of these interventions and are likely to be influenced by them. As the intervention is focused on envisioning contact and positive interaction with the refugee child, we focused on the two most obvious criteria—attitudes and contact intention toward the refugee children. We also examine these effects separately for children in lower and upper grades of elementary school. Thus, the main research question is to assess the short-term and long-term effectiveness of the imagined contact intervention conducted in lower and upper grades of elementary schools in Croatia on the change in children’s attitudes and contact intentions toward refugee children.

## 2 Materials and methods

### 2.1 Description of an imagined contact intervention

The imagined contact intervention applied in this study consisted of four sessions. Each session was conceptualized as a four-step process (based on Vezzali et al., 2020) and lasted approximately one school hour (45 min).

In the first step, the concept of refugees was discussed and clarified using a slideshow with age-appropriate definitions and photographs of children from different ethnic backgrounds. Since our participants did not have prior experience with refugees, this step was particularly important in the first session. In the following sessions, intervention facilitators made sure that children remembered the definition.

In the second step, participants were presented with a scenario describing how contact with a refuge child began and continued to imagine the contact on their own. They were encouraged to imagine a pleasant interaction (i.e., a conversation or socializing) with a refugee child. Imagined scenarios used in the intervention incorporate different ways of strengthening the effects of imagined contact in each of the four sessions—including norm-framed imagined contact scenarios (Smith and Minescu, 2022a, 2022b), scenarios with an elaborated description of the setting in which the contact takes place (e.g., Husnu and Crisp, 2010), those that include working together with an imagined child on a common goal (Kuchenbrandt et al., 2013) and scenarios in which the participant imagines to be a part of the same group as the imagined refugee child (Vezzali et al., 2015c). The scenarios were designed based on previous research and in cooperation with teachers who assessed their suitability for children of different ages. Accordingly, they were slightly different for children in the lower (second to fourth) and upper (fifth to eighth) grades of primary school, but they were designed based on the same principles. Thus, although the scenarios were not identical at the manifest level, they should have led to comparable psychological processes in children of different ages (see Table 1 for scenarios used in lower and upper grades).

**Table 1:** Content of imagined contact scenarios for children in the lower and upper grades.

Scenario for lower grades	Scenario for upper grades
Imagine that a refugee child comes to your class and that the teacher tells him or her to sit next to you. Your friends in your class are excited that you will all meet the refugee child and they encourage you to talk to him or her. At first s/he does not know what to tell you because s/he does not speak Croatian well, but you soon start to have a good time together. Soon, the bell rings, which means that the class is ending.	Imagine that a refugee your age comes to your class and that your teacher tells them to sit next to you. Your friends in your class are excited that you will all meet him/her and they encourage you to talk to the refugee. At first s/he does not know what to tell you because s/he does not speak Croatian well, but you soon start to have a good time together. Soon, the bell rings, which means that the class is ending.
Imagine you are in your favorite park. It is spring, and the weather is nice and sunny. You can hear birds singing and you can smell spring flowers. Your friends are not there today and you are playing alone. Then you notice another child your age in the park. You remember that your friends told you it was a refugee who moved to a nearby street, and who does not speak Croatian well. At the beginning, you do not know how to approach them, but you start playing and having fun together soon.	Imagine you are walking around your neighborhood. It is spring, and the weather is nice and sunny. You see neighbors passing by and cars in the street. As you come closer to your house, you notice a child your age in the street. You do not know him or her, but you have heard that he or she is a refugee who does not speak Croatian very well. You are walking towards each other, and you decide to stop and say hello. Although you are not sure what to say to each other at first, soon you start to hang out and have fun.
Imagine that it is winter and that a lot of snow fell overnight. You are happy because you will go out and make a snowman! An older neighbor in your street comes out to clear snow, and a child your age joins him and starts helping him. You recognize that it is a refugee whose family has recently moved to your street. You also decide to help your neighbor. Although the refugee child does not speak Croatian very well, the two of you are having a good time clearing the snow together.	Imagine that you are returning from school and you are walking towards your house. You see a child your age in front of you walking in the same direction. When you look at them more closely, you realize this student is a refugee who came to your school and attends another class in your school. Soon, several younger children run towards you and ask for your help to get down a ball that got stuck in a tree. Although you do not know each other, and although the refugee student does not speak Croatian very well, the two of you decide to help the children together.
Imagine that you are at a playroom for a birthday party of a friend. The room is full of balloons and toys. You notice a child you do not know, who you heard was a refugee. Soon it is time for a game, and the playroom teacher divides you into groups where you are supposed to find as many balloons of the same color as possible. You are in the group with the refugee child. At first you are not certain how it will all go because the child does not speak Croatian very well, but you soon start to look for balloons together and help each other, and you are having a great time! At the end of the game, your group has won!	Imagine you are in the school playground. It is summer, and the playground is full of children your age. There is a child your age whom you do not know, but you have heard that he or she is a refugee and that he or she does not speak Croatian very well. You are all bored and you do not know what to do. Suddenly, someone suggests that you organize a sports competition. You are in the team together with the refugee child. You introduce yourselves to each other and you start working together to be as good as possible in the game. You are helping each other and having a great time. At the end of the competition, your team has won!

Additional information on the workshops is available at: https://urn.nsk.hr/urn:nbn:hr:131:848219.

In the third step, children were asked to draw what they had imagined or describe the imagined scenario in their own words as a form of individual reinforcement of the effect of imagined contact. Methods of reinforcement also differed for children in lower and upper grades. In lower grades, children have drawn the encounter in all four sessions, followed by a short written description of the drawing. Children in upper grades have written short essays describing the contact with a refugee child during the first, second and fourth sessions, and they have drawn a comic in the third session. During this step, participants responded to four additional questions about their experience with imagining contact in each session. Questions are described in the Measures subsection.

The last step was a class discussion aimed at reinforcing the effect of imagined contact. In this step, students could volunteer to retell to the class what they had imagined or could show their drawing and explain what it depicted (for more details such as materials used and precise guidelines for the intervention implementation, see Jelić et al., 2023).

Finally, in developing the intervention, we have tried to ensure it is easy to apply in the school context. Therefore, the intervention was led by school staff instead of researchers. This was a necessary condition for the intervention to be as widely applicable as possible because some studies show that the effects of various school interventions on intergroup attitudes are significant only when conducted by researchers and not by teachers (Ülger et al., 2018), while others suggest that imagined contact interventions can be successfully applied by teachers (Vezzali et al., 2015c). Similarly, it was conducted in a group setting, i.e., all students in the class participated in the intervention simultaneously. A recent meta-analysis (Ülger et al., 2018) found greater effects when prejudice-reduction interventions were done individually than at a group level. However, the classroom is a group context by definition and interventions performed at a group level are much easier to implement in these conditions.

To sum up, the key features of this intervention, which all together represent a unique contribution to imagined contact literature, are as follows:

- It was conducted by school staff instead of researchers,
- It was conducted on a group level,
- It consisted of multiple sessions,
- It incorporated elaborated scenarios,
- It included reinforcement methods,
- It was adapted to the children’s age group.

### 2.2 Participants

A total of 1,544 children from seven Croatian primary schools that were not attended by refugee children at the time participated in the study. They were divided into an intervention group (*N*_i_ = 827 children from 48 school classes) and a comparison group (*N*_c_ = 717 children from 50 classes). Children from the lower (second to fourth; *N*_L_ = 645) and upper grades (fifth to eighth; *N*_U_ = 899) took part in the study. A total of 767 participants identified themselves as girls, 759 as boys and 18 did not state their gender. The average age of participants was M = 10.73 (SD = 2.039). Intervention and comparison groups did not differ in terms of age (*t*(1489.7) = 1.03, *p* = 0.303) or gender distribution (*χ*^2^(1) = 0.66, *p* = 0.417).

### 2.3 Procedure

The study was approved by the IRB of the Department of Psychology, Faculty of Humanities and Social Sciences, University of Zagreb, and the data collection procedures were conducted from September 2021 until February 2022. All measures used in the study were piloted on a sample of 231 children from the second, third, fourth, sixth, and eighth grades from a school that was not included in the main study. We also wanted to ensure that all children understood the items in the questionnaire, so all measures were refined based on the quantitative data and qualitative feedback from two focus groups of children from the second and third grades.

Before the intervention started in the treatment group, participants from both the intervention and comparison groups filled out a paper-and-pencil questionnaire with the measures described in the Instruments chapter. The testing was carried out in a group setting within the existing classes. Before filling out the questionnaire, the researchers made sure that the children knew who the refugee children were and gave them a uniform, age-appropriate explanation. On top of that, psychology students accompanied the researchers to help with the distribution of questionnaires and to offer additional individual explanations when necessary.

Following the pre-test, four intervention sessions were carried out in existing classes by trained members of the school staff. Intervention facilitators have received handbooks with a description of the theoretical basis of the intervention and detailed preparation for each of the sessions, as well as guidelines for reacting in potentially challenging situations (if the child refuses to imagine, imagines a negative contact with a refugee child, if no one wants to share what they imagined with the class, etc.). These guidelines were prepared in consultation with teachers of the schools involved in the development of the intervention. Furthermore, each class that is part of the intervention group had its own set of worksheets, in which the intervention facilitator wrote down notes about children’s reactions and important events during the implementation.

In line with the results of the recent meta-analysis (Miles and Crisp, 2014), which show that the effect of the imagined contact does not depend on whether the control group has an alternative task or not, the comparison group did not engage in any specific task.

After the intervention, a post-test and a follow-up assessment were conducted in both intervention and comparison groups. The dates of each measurement occasion were recorded since we expected some variation in time lags between classes due to the COVID-19 pandemic and school scheduling conflicts. The first post-test was administered, on average, 6 days after the last session (range from 0 to 38 days), while the follow-up assessment took place on average 82 days after the last session (range from 63 to 118 days). To ensure consistency, post-tests in the comparison classes were coordinated with those in the intervention classes.

### 2.4 Measures

Since the analyses were performed on latent factors to account for the measurement error, the following measures were used as indicators of two factors or outcomes of interest: intergroup attitudes and contact intentions.

*Intergroup attitudes*. The latent factor of intergroup attitudes was based on three indicators:

*Positive stereotypes about refugee children*. A measure of positive stereotypes consists of three positive adjectives (polite, tidy, and friendly; adapted based on Cameron et al., 2006). Participants rated how much they agreed that each of the described characteristics applies to refugee children on a scale of 1 (*not at all*) to 5 (*completely*), and a total score was calculated as an average of the three items.

*Negative stereotypes about refugee children*. A measure of negative stereotypes consists of three negative adjectives (lazy, unkind, and mean; adapted based on Cameron et al., 2006). Participants rated how much they agree that each of the described characteristics applies to refugee children on a scale of 1 (*not at all*) to 5 (*completely*). A total score was calculated as an average of the three items.

*General evaluation of refugee children*. Attitude was further assessed using a single item asking participants for their general opinion of refugee children on a scale of 0 (*very bad*) to 10 (*very good*).

*Intentions for contact with refugee children*. To measure the latent contact intentions factor, we used a translation and adaptation of three items previously used in similar studies (Cameron et al., 2006; Vezzali et al., 2012b), which served as indicators. The children answered how they would act if they met a refugee child they did not know—would they approach him/her and get to know him/her, would they hang out, and would they go for ice cream together? The participants expressed their agreement on a scale of 1 (*not at all*) to 5 (*completely*). For each of the items, a higher score indicates a greater intention to contact refugee children in future.

In addition, participants provided information on sociodemographic data and their experiences during the imagined contact tasks. In each of the four sessions, children from the intervention group provided ratings of the ease of imagining the encounter (*I found it easy to imagine meeting a refugee child*), the positivity (*I have imagined hanging out nicely with a refugee child*) and level of detail (*I have imagined hanging out with a refugee child in detail*) in their imagined scenarios, and how much they enjoyed the task (*I liked this task*), on a scale from 1 to 5. Averages across all sessions were calculated for each participant and each of the four characteristics of the imagined experience.

### 2.5 Statistical analysis method

Six participants were excluded from further analysis, either because they did not participate in any of the imagined contact sessions or because they had issues understanding the questionnaire. One additional participant was excluded from the analyses concerning contact intentions since they did not answer any item related to that construct. Therefore, the final sample for analyses conducted from this point onward consisted of 1,538 participants, with the outcome being intergroup attitudes and 1,537 for contact intentions. Descriptive statistics for children in the lower and upper grades are shown in Table 2. All analyses were conducted in R (version 4.1.1; R Core Team, 2021).

**Table 2:** Descriptive statistics on manifest variables in the intervention and comparison group through three measurement points for children in lower and upper grades separately (total *N* = 1,538).

Construct	Lower grades (*N* = 642)						Upper grades (*N* = 896)					
	Comparison (*N* = 297)			Intervention (*N* = 345)			Comparison (*N* = 420)			Intervention (*N* = 476)		
	*N*	*ω*	M (SD)	*N*	*ω*	M (SD)	*N*	*ω*	M (SD)	*N*	*ω*	M (SD)
Attitudes												
Positive stereotypes T1	285	0.65	3.73 (0.83)	329	0.57	3.96 (0.80)	400	0.73	3.63 (0.72)	454	0.74	3.65 (0.73)
Positive stereotypes T2	278	0.76	3.64 (0.96)	328	0.69	3.98 (0.84)	394	0.76	3.71 (0.77)	440	0.77	3.74 (0.78)
Positive stereotypes T3	292	0.78	3.66 (0.97)	331	0.78	3.94 (0.87)	403	0.77	3.67 (0.78)	463	0.81	3.67 (0.83)
Negative stereotypes T1	284	0.59	1.92 (0.88)	330	0.62	1.75 (0.81)	400	0.63	1.95 (0.71)	454	0.68	1.97 (0.75)
Negative stereotypes T2	278	0.72	1.91 (0.92)	328	0.74	1.76 (0.93)	394	0.71	2.04 (0.76)	438	0.74	2.06 (0.78)
Negative stereotypes T3	292	0.78	1.96 (0.99)	331	0.74	1.76 (0.85)	402	0.78	2.05 (0.78)	463	0.76	2.03 (0.81)
General evaluation T1	281	–	7.21 (2.33)	323	–	7.50 (2.21)	399	–	7.24 (1.9)	453	–	7.17 (1.84)
General evaluation T2	269	–	6.99 (2.41)	320	–	7.88 (2.10)	390	–	7.33 (1.89)	439	–	7.26 (2.12)
General evaluation T3	289	–	6.79 (2.70)	329	–	7.83 (2.22)	400	–	7.16 (1.94)	460	–	7.20 (2.12)
Contact intentions												
T1	285	0.89	3.42 (1.32)	331	0.86	3.72 (1.21)	395	0.91	3.38 (1.21)	452	0.92	3.44 (1.15)
T2	277	0.93	3.25 (1.41)	328	0.86	3.86 (1.18)	393	0.92	3.30 (1.21)	435	0.92	3.32 (1.26)
T3	292	0.91	3.32 (1.34)	331	0.86	3.74 (1.16)	400	0.93	3.30 (1.20)	459	0.95	3.21 (1.30)
Children’s experience												
Ease of imagining	–	–	–	340	–	4.16 (0.87)	–	–	–	476	–	3.61 (1.06)
Positivity	–	–	–	341	–	4.68 (0.58)	–	–	–	476	–	4.26 (0.90)
Level of detail	–	–	–	341	–	4.05 (0.87)	–	–	–	476	–	3.43 (0.95)
Enjoyment	–	–	–	341	–	4.44 (0.89)	–	–	–	476	–	3.50 (1.19)

All measures had a five-point rating scale, apart from the measure of general evaluation which had a theoretical range from 0 to 10. For contact intentions averages are shown across the three items. Information about children’s experience during the imagined contact task is available only for the intervention group. T1, pre-test; T2, post-test; T3, follow-up.

Multigroup latent change score analyses (McArdle and Prindle, 2008; McArdle, 2009) were used to compare the change in intergroup attitudes and contact intentions between the intervention and comparison groups separately for children in lower and upper grades. Latent change variables have their own mean (i.e., intercept) and variance, providing information about the average change in the sample and whether these changes occur equally for all participants (McArdle, 2009). Latent change scores used in this study were derived from latent factors, ensuring that they capture differences without the accumulation of measurement error (McArdle, 2009; Cáncer et al., 2021; Matusik et al., 2021). Analyses were conducted using package *lavaan* (Rosseel, 2012).

For each of the outcome variables in this study, two latent variables describing change were used—first, a variable describing change between the pre-test and post-test (ΔT1T2), which represents a more immediate effect of an intervention, and second, a variable describing change between the pre-test and a delayed follow-up (ΔT1T3), representing a more long-term effect. Furthermore, we controlled for the effects of the baseline level on the latent changes by including proportional change parameters (McArdle and Prindle, 2008; McArdle, 2009). The same model was estimated in the intervention and comparison group. Differences in the model parameters were examined by constraining them to be equal across groups and comparing the fit of the unconstrained and constrained models with a Satorra–Bentler scaled chi-square difference test. Effect sizes were computed for each of the eight combinations of the variables (attitudes vs. contact intentions, younger vs. older children, short-term vs. delayed change). If the variances of change scores in intervention and comparison groups did not differ significantly, Cohen’s *d* was calculated based on the latent change scores. However, if the variances of the two groups differed significantly, Glass’ delta was calculated.

## 3 Results

### 3.1 Preliminary analyses

We compared participant’s experience during the intervention between the lower and upper grades. The results showed that children from the lower grades reported it was easier for them to imagine contact with a refugee child (*t*(797.10) = 8.17, *p* < 0.001), their imagined interactions were perceived as more positive (*t*(807.54) = 8.12, *p* < 0.001) and detailed (*t*(766.78) = 9.62, *p* < 0.001), and they enjoyed the activity more (*t*(813.52) = 12.90, *p* < 0.001) than children from the upper grades (see Table 2 for average results).

Robust maximum likelihood and full-information maximum likelihood were used in the main analysis to account for the absence of multivariate normality and handle missing data. In addition, we calculated intraclass correlation coefficients (ICCs) using the *lme4* (Bates et al., 2015) and *misty* (Yanagida, 2023) packages. While the majority of the ICC values were small, several indicators exhibited ICCs greater than 0.10. These indicators were few, but they were found in both the younger and older subsample, in all three time-points and in both the intervention and comparison groups. The highest ICC value was 0.18, which suggests that 18% of the variance in the said indicator can be attributed to classroom membership. While this indicates that children who share a classroom have somewhat more similar attitudes than children from other classes, ICC values did not reach excessively high levels (>0.30; McNeish et al., 2017). Since we focused primarily on the effectiveness of an intervention on an individual level in this study, we decided to use clustered standard errors to accommodate the nested data structure (McNeish et al., 2017).

Finally, to scale the latent factors, the mean and variance of the baseline in the comparison group were set to zero and one, respectively. Scalar invariance across the two groups (intervention and comparison) and the three time-points was established both for attitudes and contact intentions and separately for different age groups. Model fit evaluations were based on *χ*^2^, CFI, RMSEA and its 90% confidence interval, and SRMR, while model comparisons employed criteria for large samples with equal sample sizes, as proposed by Chen (2007).

### 3.2 Main analysis

In the text below, we focus on the differences in intercepts of latent change factors, which indicate the short- and long-term effectiveness of an intervention.

#### 3.2.1 Intergroup attitudes

**Figure 1:**
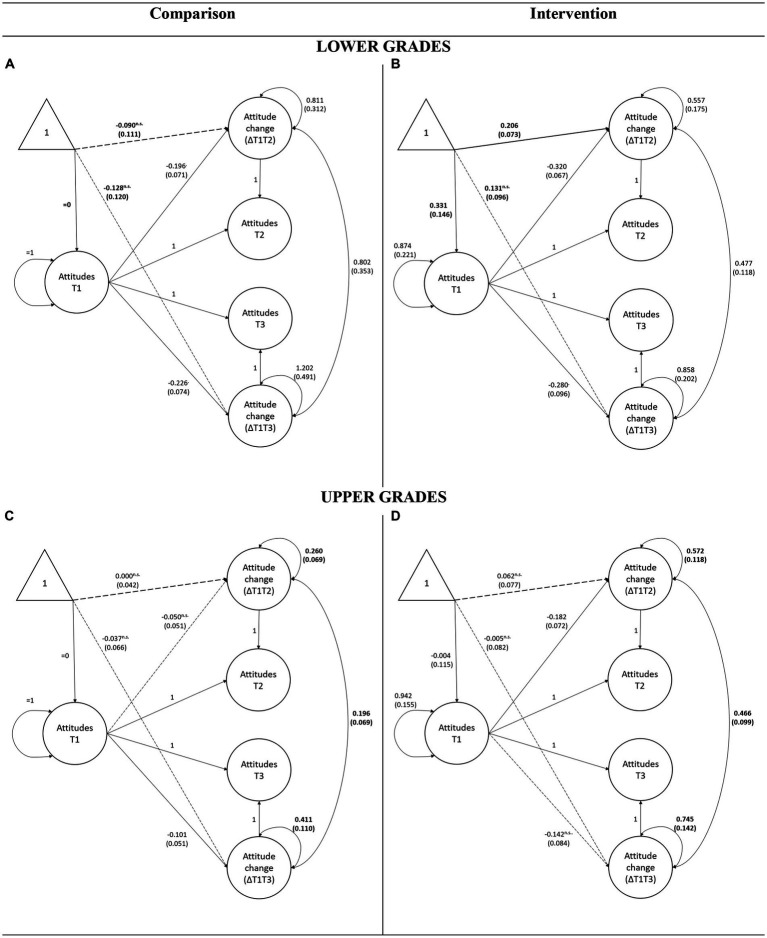
Multigroup LCS model illustrating change in intergroup attitudes between the comparison and intervention group for lower and upper grades separately. Unstandardized parameters are presented, with dashed arrows representing paths that do not differ from zero. Standard errors are indicated within brackets, and parameters that significantly differ between groups are shown in bold.

In the lower grades, the multigroup model allowing the groups to differ in change scores fits the data well (*χ*^2^(50) = 51.97, *p* = 0.397, CFI = 1.000, RMSEA = 0.000 [0.000–0.042], SRMR = 0.039). The unconstrained multigroup model is shown in Figures 1A,B, and all parameters which differed significantly across the two groups in a series of constrained model comparisons are shown in bold.

The results indicate that the intercept of short-term change in attitudes was positive and significantly different from zero in the intervention group (ML estimate = 0.206, std. error = 0.073, *p* = 0.005). In the comparison group, the intercept of short-term change is negative (ML estimate = −0.090, std. error = 0.111, *p* = 0.417) but not significantly different from zero. Constraining the change scores describing the short-term change to be equal across intervention and comparison groups suggested that they differ significantly (Δ*χ*^2^(1) = 10.52, *p* = 0.001). The short-term change in intergroup attitudes was more positive in the intervention than in the comparison group. Cohen’s d calculated on the latent change scores was *d* = 0.36.

Focusing on the delayed effect of the intervention, the intercept of the long-term change in attitudes in the intervention group was positive but not significantly different from zero (ML estimate = 0.131, std. error = 0.096, *p* = 0.171), while an intercept of the same change in the comparison group was negative and also not significantly different from zero (ML estimate = −0.128, std. error = 0.120, *p* = 0.288). However, constraining the two intercepts to be equal across groups yielded a significant chi-square difference test (Δ*χ*^2^(1) = 5.61, *p* = 0.018), suggesting that intervention had significant long-term effects on the attitudes of the children in the lower grades of primary school. Cohen’s *d* calculated on the latent change scores was *d* = 0.26. Significant differences in other parameters are depicted in Figure 1.

In the upper grades, the unconstrained model also had a good fit to the data (*χ*^2^(50) = 78.04, *p* = 0.007, CFI = 0.992, RMSEA = 0.039 [0.019–0.056], SRMR = 0.037), and the parameters of the model are shown in Figures 1C,D.

The results indicate that none of the latent changes have an intercept, which significantly differs from zero for either of the groups. Furthermore, multigroup comparisons suggest there are no significant differences between the intervention and comparison groups, both in short- (Δ*χ*^2^(1) = 0.51, *p* = 0.474) and long-term (Δ*χ*^2^(1) = 0.10, *p* = 0.752). Glass’ deltas were Δ = 0.12 and Δ = 0.05 for the short- and long-term change, respectively. However, variances of latent changes are significantly larger in the intervention group (Δ*χ*^2^(1) = 9.09, *p* = 0.003 for short-term and Δ*χ*^2^(1) = 8.09, *p* = 0.004 for long-term change). This suggests that different children who participated in the intervention experienced different amounts of change.

Overall, the analyses suggest that an imagined contact intervention leads to a positive short-term and long-term change in intergroup attitudes, but only for children in the lower grades of primary school.

#### 3.2.2 Contact intentions

**Figure 2:**
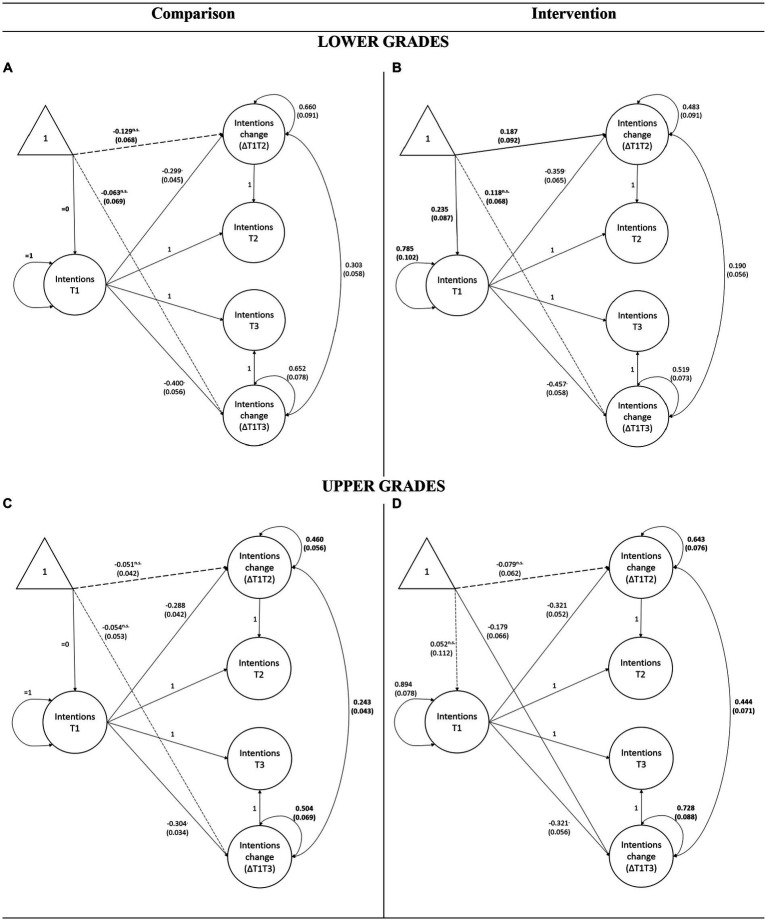
Multigroup LCS model illustrating change in contact intentions between the comparison and intervention group for lower and upper grades separately. Unstandardized parameters are presented, with dashed arrows representing paths that do not differ from zero. Standard errors are indicated within brackets, and parameters that significantly differ between groups are shown in bold.

In the lower grades, an unconstrained multigroup model had a good fit to the data (*χ*^2^(50) = 70.66, *p* = 0.029, CFI = 0.994, RMSEA = 0.037 [0.000–0.059], SRMR = 0.037). The unconstrained multigroup model for contact intentions is shown in Figures 2A,B. As with the intergroup attitudes, an intercept of short-term change in contact intentions was positive and significantly different from zero in the intervention group (ML estimate = 0.187, std. error = 0.092, *p* = 0.042) and negative but not significantly different from zero in the comparison group (ML estimate = −0.129, std. error = 0.068, *p* = 0.058). Constraining the two change scores to be equal across groups indicated that short-term change in contact intention was more positive in the intervention group (Δ*χ*^2^(1) = 10.59, *p* = 0.001). Cohen’s *d* calculated on the latent change scores was *d* = 0.42.

The intercept of the long-term change in contact intentions was positive but not significantly different from zero in the intervention (ML estimate = 0.118, std. error = 0.068, *p* = 0.086) and negative but not significantly different from zero in the comparison group (ML estimate = −0.063, std. error = 0.069, *p* = 0.365). However, constraining the intercepts of long-term change to be equal across groups suggested that intervention had significant long-term effects on the contact intentions of the children in the lower grades of primary school (Δ*χ*^2^(1) = 4.63, *p* = 0.031). Cohen’s *d* calculated on the latent change scores was *d* = 0.24.

In the upper grades, the unconstrained model fits the data well (*χ*^2^(50) = 80.49, *p* = 0.004, CFI = 0.995, RMSEA = 0.040 [0.021–0.057], SRMR = 0.025), and it is shown in Figures 2C,D. The results indicate that intercepts of all latent changes in both intervention and comparison groups were negative, indicating a reduction in contact intentions. However, only an intercept of long-term change in the intervention group was significantly different from zero (ML estimate = −0.179, std. error = 0.066, *p* = 0.007). Nevertheless, multigroup comparisons suggest there are no significant differences between the intervention and comparison groups, either in the short-term change (Δ*χ*^2^(1) = 0.16, *p* = 0.690) or in the long-term change (Δ*χ*^2^(1) = 3.15, *p* = 0.076). Glass’ deltas were Δ = −0.04 and Δ = −0.18 for the short- and long-term change, respectively. As was the case with intergroup attitudes, variances of latent changes are significantly larger in the intervention group (Δ*χ*^2^(1) = 8.06, *p* = 0.005 for short-term and Δ*χ*^2^(1) = 6.32, *p* = 0.012 for long-term change). In line with the results on the measures of intergroup attitudes, imagined contact intervention also leads to positive short-term and long-term changes in contact intentions. However, once again, this effect is present only in children in the lower grades.

#### 3.2.3 Additional analyses

In addition to the main findings, the analyses offer information on each of the LCS model parameters, which can be interpreted using Figures 1, 2. For example, incorporating regressions of change scores on the baseline in all analyses allowed us to control for the initial differences in levels of attitudes and contact intentions shown in T1. Figures 1, 2 also show that these effects of the baseline were predominantly negative (i.e., children with more positive initial attitudes have exhibited less increase), and the regression coefficients did not differ between the groups (i.e., the baseline predicted change equally in intervention and comparison group, suggesting there was no interaction).

## 4 Discussion

Previous research showed that preparatory interventions that could help facilitate the integration and adaptation of refugee children to their new schools are needed, especially in countries with no prior experience with refugee integration (Vrdoljak et al., 2024). Our aim of this study was to assess the short-term and long-term effectiveness of the imagined contact intervention conducted in lower and upper grades of elementary school on the change in children’s attitudes and contact intentions toward refugee children. We designed an imagined contact intervention in a way that tried to respond to previously established obstacles (Husnu and Crisp, 2010; Miles and Crisp, 2014; Vezzali et al., 2015c; Ülger et al., 2018; Smith and Minescu, 2022a, 2022b; Vrdoljak et al., 2023) that could be the reason why the intervention is sometimes effective in changing attitudes toward outgroup members, and sometimes not. Specifically, we tried to amplify the vividness of the imagined interaction in intervention by developing modified scenarios, using multiple sessions, and incorporating reinforcement techniques after imagining positive interaction with the refugee child.

Results of this study show that the intervention had a significant positive short-term and long-term impact on intergroup attitudes and contact intentions for children in the lower grades, meaning that it effectively improved their attitudes toward refugees. This suggests that engaging younger pupils (up to the age of 11) in imagining positive encounters with refugee children can immediately impact their attitudes and contact intentions in a positive way. Furthermore, we also confirmed the potential of this intervention to foster long-term attitude change and thus cultivate a more inclusive and accepting school environment. This is particularly important since the follow-up period used in this study was much longer than previous successful imagined contact interventions (e.g., Stathi et al., 2014; Vezzali et al., 2015c, 2020; Ginevra et al., 2021; Smith and Minescu, 2022b). Since imagined contact is fairly easy to implement in the school context, especially in contexts which have only a small community of outgroup members, intervention effects lasting up to two and a half months are particularly encouraging for future use in prejudice-reduction programs. However, the intervention did not have a significant effect on changing the attitudes and contact intentions of the upper-grade students.

While the influence of imagined contact on children’s intergroup attitudes and contact intentions has been observed quite consistently in prior experimental research (e.g., Stathi et al., 2014; Vezzali et al., 2015b, 2020; Ginevra et al., 2021), some studies have not found the expected effects, especially with the age groups which correspond to upper grades in our study (11 years or older; e.g., Fleva, 2014; Mazure, 2016; Kinghan, 2019). Therefore, these findings are not entirely surprising, and they imply that the effectiveness of the intervention may be influenced by the age or developmental stage of the children. In addition, for both outcome measures, older children who participated in the intervention showed more variability in the changes than those who did not. This suggests that different children possibly responded to the intervention in different ways, and potential moderators of the intervention effectiveness could shed additional light on these results. These could include, for example, prevention goals (participants who focus on covering their prejudice during imagined contact could respond negatively to the task; West and Greenland, 2016), previous intergroup contact (Hoffarth and Hodson, 2016) or perceived difficulty of imagined contact task (West and Bruckmüller, 2013).

In addition, the experiences of children in the lower and upper grades differed. Younger children found it easier to imagine intergroup interaction; they perceived the interaction as more positive and detailed and enjoyed the task more. All of these characteristics of the imagined experience could potentially lead to larger effects (Husnu and Crisp, 2010; West et al., 2011; West and Bruckmüller, 2013). Differences in the participant’s reactions to the imagined contact task could stem from factors related to the child’s development, or the differences in the tasks themselves.

Overall, our findings highlight the importance of considering both methodological and developmental factors underlying age differences in the effectiveness of the intervention. Considering age differences in the effectiveness of the intervention, one possibility is that younger children exhibit a greater openness to various interventions within the school context. Developmentally, they are not yet in the rebellious years and tend to show less resistance toward authority figures. In other words, younger pupils possess a higher level of respect for the authority of their teachers, and they are more inclined to follow instructions and participate in various activities with enthusiasm. This is also evident from the greater enjoyment they reported during the intervention. Moreover, their malleable attitudes may stem from their limited understanding of complex concepts, such as refugees. As their comprehension of these subjects is still developing, it becomes easier to shape and change their attitudes through education and guidance. Overall, the general receptiveness and willingness of younger children make them more receptive to interventions in schools than adolescents.

Another possibility is that younger children are more susceptible to the authority of the teacher who carried out the intervention because, in their case, it was the teachers who are central figures in their schooling, who normally teach them most of the school subjects, who know them well, and who in this sense can more easily establish authority in the class. Adolescents are already in subject classes, and the intervention in the upper grades was led by the teachers of one of the subjects, whom they see only a few times a week and do not necessarily develop strong ties with, as is the case with younger children and their class teacher. Teachers who conduct an intervention communicate their own positive attitude toward refugee children and shape a positive teacher norm regarding contact with refugees. If younger children are closer to their teachers, their perception of the teacher’s positive stance on refugees could contribute to the effectiveness of the intervention (Jones and Rutland, 2018). Conversely, if children in the upper grades do not feel as close to the teacher, they could dismiss the intervention more easily.

On the other hand, as stated earlier, younger and older children did not receive completely identical interventions, so these differences in the procedure might also be the reason why the intervention was more effective for younger children. For instance, we aimed to ensure psychological similarity of imagined scenarios for children in lower and upper grades, rather than focusing on providing identical stories. The scenarios were quite similar, created based on the same theoretical criteria and inspected and approved by primary school teachers. Nonetheless, it is still possible that the scenarios used with children in lower grades were more effective in establishing the intervention effects.

Furthermore, the reinforcement methods used were different depending on the age group, with younger children drawing their imagined interaction with the refugee child, whereas older children had three sessions in which they were writing a short essay and only one session in which they were drawing a cartoon. It is possible that drawing is a more engaging activity for all children, regardless of their age, because it is less like other activities for which they are usually evaluated in schools (such as writing). In fact, teachers’ qualitative feedback collected during the intervention suggests that the writing task used as a form of reinforcement in upper grades was met with more apprehension than the drawing task used in lower grades, which is corroborated by children’s quantitative feedback. This apprehension could be related to the lower levels of enjoyment reported by older children during the task and may have had a negative influence on the study results. Children who did not enjoy the intervention were likely less inclined to imagine a positive interaction and provide detailed scenarios, both of which have been found to impact the effectiveness of imagined contact (Miles and Crisp, 2014). So, if the choice of the reinforcement method plays an important role in the effectiveness of interventions, it is essential to engage children in activities that are well-suited to their developmental stage but are also interesting enough. Furthermore, it is possible that it is generally easier to draw the imagined experience than to describe it in words or that drawing increases the vividness of the representation of an imagined interaction in the memory more effectively, all of which could contribute to different perceptions of the activities by children in the lower and upper grades (Husnu and Crisp, 2010). In that sense, incorporating drawing instead of writing can make the activities more interactive, effective, and enjoyable for all children, thus increasing their participation and understanding. Further research is needed to explore potential factors contributing to the varying effects observed between different grade levels.

Overall, the findings of this study highlight the effectiveness of imagined contact intervention in shaping positive attitudes toward refugees among elementary school children, with both immediate and sustained impact. These conclusions support the continued implementation of such interventions in educational settings to promote inclusivity and the importance of introducing interventions targeting attitudes toward refugees in an age-appropriate manner to achieve maximum effectiveness.

### 4.1 Limitations and suggestions for future studies

Several limitations of this study need to be mentioned. First, in order to compare the results of younger and older children, we used Likert-type scales to assess their explicit attitudes and contact intentions. Our measures have been pre-tested to ensure they are age appropriate, but future studies could also benefit from including visual analog scales with children. Similarly, the field could benefit from more thorough exploration of imagined contact’s effects on children’s automatic (implicit) prejudice. Implicit attitudes are generally regarded as less susceptible to demand characteristics (Greenwald and Banaji, 1995). They are only weakly correlated with explicit measures (Hofmann et al., 2005), have different developmental pathways (Baron and Banaji, 2006; Degner and Wentura, 2010), and differently predict behaviors (Rydell and McConnell, 2006). Only one study examined and detected the effects of imagined contact on implicit attitudes using an Implicit Association Test (Vezzali et al., 2012a). However, different patterns of automatic prejudice development are found using measures, which do not depend on forced categorization, such as affective priming tasks (Degner and Wentura, 2010), and studies focusing on imagined contact’s influence on these measures are particularly needed.

Furthermore, this study aimed to explore the effectiveness of imagined contact in realistic conditions, where the intervention was regarded as a part of the regular school activities. This increased the ecological validity of the findings, but it has also led to variability in the time elapsed between the intervention and post-intervention measurements.

Since this study employed somewhat different approaches in lower and upper grades, it is challenging to determine whether the characteristics of the children (developmental stage), the intervention (facilitator role, differences in scenarios, and reinforcement techniques), or their interaction are accountable for the age differences. For example, it is possible that older children quickly get bored by repetitive activities, while younger children need more repetitions in order to consolidate the imagined experience. Therefore, a more systematic examination of age differences, where intervention procedures are the same for younger and older children, is needed. Furthermore, these procedures could be varied in order to establish the optimal way in which imagined contact can be employed with different age groups.

Nevertheless, imagined contact proved to be a useful tool to reduce prejudice in children and thus pave the way for positive future interactions and successful integration in a context where the number of refugees is small, and opportunities for contact are scarce. However, the researchers and practitioners should bear in mind that the effectiveness of the imagined contact intervention depends on choosing the age-appropriate approach/method. Future studies should consider developing more efficient imagined contact interventions specifically tailored for adolescents. This age group may require different approaches to maintain engagement and motivation. One potential approach when the intervention consists of several sessions could be making the later sessions more impressionable according to their age and interests to avoid repetition and fatigue. Additionally, diversifying the sessions, such as incorporating drawing comics and role-playing activities, could further enhance the effectiveness of the interventions for adolescents. In summary, further analysis is required to better understand these differences and tailor interventions accordingly to suit the specific needs and cognitive development of children at different grade levels.

Finally, it is also possible that children in the upper grades could profit more from activities which include more substantial discussions on matters of prejudice and discrimination. Indeed, imagined contact could also be used as a part of more complex interventions that would be based on different approaches to reducing children’s prejudices. It is thus possible to integrate the imagined contact with multicultural curriculum or anti-prejudice programs. Instead of just passively learning about cultural differences, tolerance and prejudice, students could imagine that they learn information about the country that a refugee child comes from through interaction with a refugee child, or they could imagine talking about how it feels to be discriminated against based on refugee status (Crisp and Turner, 2012). These recommendations are along the lines of a recent study by Ginevra et al. (2021), who combined imagined contact with specific instructions on how to act during initial contact situations. This intervention provided children with specific and detailed information on what to expect and how to communicate with children with developmental disabilities before taking part in imagined contact, which led to improved attitudes and contact intentions among the majority of children compared to using imagined contact on its own. Future studies could further examine the potential for incorporating imagined contact in more complex intervention programs.

## 5 Conclusion

In conclusion, the study suggests that the intervention had positive and lasting effects on shaping intergroup attitudes and contact intentions in the lower grades but did not yield significant changes in these measures for students in the upper grades of elementary school. These findings highlight the importance of considering the age and developmental stage of students and the methods of reinforcement of the imagined contact effects when designing interventions to promote positive intergroup attitudes in educational settings. Further analysis is required to better understand these differences and tailor interventions accordingly to suit the specific needs and cognitive development of children at different grade levels.

## Data availability statement

The raw data supporting the conclusions of this article will be made available by the authors, without undue reservation.

## Ethics statement

The studies involving humans were approved by the Institutional Review Board of the Department of Psychology, Faculty of Humanities and Social Sciences, University of Zagreb. The studies were conducted in accordance with the local legislation and institutional requirements. Written informed consent for participation in this study was provided by the participants’ legal guardians/next of kin.

## Author contributions

AV: Writing – original draft, Visualization, Project administration, Methodology, Investigation, Formal analysis, Data curation. DB: Writing – review & editing, Methodology, Investigation, Funding acquisition, Conceptualization. NS: Writing – review & editing, Methodology, Investigation. RF: Writing – review & editing, Methodology. FB: Writing – review & editing, Methodology, Funding acquisition, Conceptualization. MJ: Writing – original draft, Supervision, Project administration, Methodology, Investigation, Funding acquisition, Conceptualization.
